# Signal Partitioning Algorithm for Highly Efficient Gaussian Mixture Modeling in Mass Spectrometry

**DOI:** 10.1371/journal.pone.0134256

**Published:** 2015-07-31

**Authors:** Andrzej Polanski, Michal Marczyk, Monika Pietrowska, Piotr Widlak, Joanna Polanska

**Affiliations:** 1 Institute of Informatics, Silesian University of Technology, Gliwice, Poland; 2 Institute of Automatic Control, Silesian University of Technology, Gliwice, Poland; 3 Maria Sklodowska-Curie Memorial Cancer Center and Institute of Oncology, Gliwice, Poland; Indiana University, UNITED STATES

## Abstract

Mixture - modeling of mass spectra is an approach with many potential applications including peak detection and quantification, smoothing, de-noising, feature extraction and spectral signal compression. However, existing algorithms do not allow for automated analyses of whole spectra. Therefore, despite highlighting potential advantages of mixture modeling of mass spectra of peptide/protein mixtures and some preliminary results presented in several papers, the mixture modeling approach was so far not developed to the stage enabling systematic comparisons with existing software packages for proteomic mass spectra analyses. In this paper we present an efficient algorithm for Gaussian mixture modeling of proteomic mass spectra of different types (e.g., MALDI-ToF profiling, MALDI-IMS). The main idea is automated partitioning of protein mass spectral signal into fragments. The obtained fragments are separately decomposed into Gaussian mixture models. The parameters of the mixture models of fragments are then aggregated to form the mixture model of the whole spectrum. We compare the elaborated algorithm to existing algorithms for peak detection and we demonstrate improvements of peak detection efficiency obtained by using Gaussian mixture modeling. We also show applications of the elaborated algorithm to real proteomic datasets of low and high resolution.

## Introduction

Current computational methodology for processing signals from spectra registered by mass spectrometry (MS) in mixtures of proteins and/or peptides usually involves sequences of signal processing operations organized in a manner leading to the detection and quantification of spectral peaks. When proteomic mass profiles are analyzed and interpreted, spectral peaks are used as features of MS spectra. It is assumed that each spectral peak corresponds to a certain peptide/protein species, and the composition of the mass spectrum carries direct information on composition of the analyzed samples. Currently, there are already more than a dozen algorithms-, either publicly available or as commercial software packages, that enable proteomic MS spectral peak detection and quantification [1–17]. Different algorithms apply different procedures, a different order and/or variants of signal processing operations. Algorithms can also differ with respect to types of proteomic data (e.g. MALDI/SELDI-ToF profiling, MALDI-IMS, LC-MS/MS).

A potentially useful approach to computational processing of proteomic MS is modeling spectral signals by mixtures of component functions. Some results in this area were published in [18–24]. A natural choice for the component functions are Gaussian distribution functions. However, the use of other component functions has also been studied [18]. Several advantages of using mixture modeling for protein MS spectra are highlighted in the referenced studies [18–24]. Using mixture models potentially allows for more accurate peak detection and quantification. In particular, in the cases where there are overlaps between components (peaks), mixture models enable detecting components “hidden” behind others. Components of mixture models of MS spectra are characterized by both positions and shapes (widths), while in most peak detection methods the information on shapes is missing. Fitting a mixture of components model to actual MS spectra allows for achieving higher sensitivity in detecting peaks of low intensity. The method of decomposition of the spectral signal into components can be more robust against disturbances.

Applications of mixture modeling to proteomic MS spectra were researched in [18–24] by analyzing proteomic actual mass spectra, or their fragments, and by conducting experiments involving fitting mixture models to data. A computational model and some exemplary results were presented in [22]. In [19] Kempka and coauthors studied a model based on the biophysical mechanisms of forming peaks in the MALDI ToF MS spectrum, with two Gaussian components corresponding to two sets of ions formed during the peptide ionization stage. They have demonstrated improvement of estimation of m/z of peptide ions by using expectation of the higher-narrower component in the mixture. Dijkstra and coauthors [18] have proposed an algorithm for fitting a mixture of a uniform distribution, exponential distribution and a number of log normal distributions to SELDI ToF spectra. Wang and coauthors [24] fitted a mixture of polynomial and Gaussian components to fragments of SELDI ToF spectra and used the MCMC (Markov chain Monte Carlo) approach for iterative estimation of mixture parameters. Noy and Fasulo [20] proposed a method of decomposing protein mass spectra with a set of component distributions derived from peptide models expected to be present in the samples. Positions and shapes of Gaussian components were fixed, the model was fitted to the data by iterations involving only component weights (Watson—Nadaraya iterations). Pelikan and Hauskrecht [21] also used predefined components following from characteristics of peptides/proteins expected in the samples. They have fitted model to data by using Bayesian probabilistic model and dynamic programming algorithm. In a recent paper [23] the authors have fitted homoscedastic Gaussian mixture models to small fragments of high resolution spectra and demonstrated its efficiency for MS signal quantification.

While highlighting a potential of application of mixture modeling to proteomic mass spectra, the studies mentioned above did not lead to algorithms capable to perform analyses based on automated mixture decompositions at the whole spectrum scale. Methods presented in [20] and [21] enable whole spectrum analyses, but require collecting information on peptides expected in the samples, which results in the restriction of its application to analyses of samples with a known peptide composition. The methods presented in [18], [19] and [23] could be applied to analyses at the whole spectrum scale only with a large amount of human processing involving e.g., the appropriate partitioning of spectral lines. Consequently, in the referenced studies the algorithms for modeling proteomic MS signals by mixtures of component functions were not compared to existing algorithms and software packages for peak detection in the sense of performing sufficiently large computational experiments and involving e.g., comparisons of sensitivities/specificities of peak detection, accuracy of estimation of m/z etc.

In this paper we present a new algorithm for the Gaussian mixture modeling of protein mass spectra based on partitioning the MS signal into smaller fragments. The fragmented spectra are separately decomposed into mixture models. The obtained parameters of components for all fragments are then aggregated and used as the mixture model of the whole spectrum. The main idea of partitioning the MS signals into fragments by using “splitters”, as well as other ideas of the elaborated algorithm, are described in detail in the “Methods” section of this paper. Partitioning the MS signal into fragments allows for overcoming obstacles encountered in the previous studies. Both initializing and executing EM iterations is much easier for the smaller fragments of the MS signal than for the whole spectrum. Another advantage of partitioning the MS signals is the possibility of parallelizing the computations. Partition of the spectral signals into fragments is augmented by the use of an existing algorithm for peak detection for proteomic MS spectra.

We verify efficiency of the developed algorithm. In the first step of verification of our methodology we use our algorithm as a tool for improving peak detection in simulated mass spectra. We present comparisons of our algorithm of peak detection to the two peak detection algorithms of high efficiency published in the literature, MassSpecWavelet (based on continuous wavelet transform, CWT, approach) [3] and Cromwell (based on spectra differentiation) [2]. Comparisons are based on a large number of artificially generated datasets. We demonstrate the improvements achieved by using Gaussian mixture modeling. In the second step of verification of the methodology we show Gaussian mixture decompositions of real proteomic datasets [25, 26, 27]. For the Aurum dataset [27] we demonstrate the improvement of the accuracy of estimation of positions of peaks by using GMM. For datasets from [25, 26] we highlight abilities of GMM modeling method to detect hidden spectral components to represent skewed shapes of spectral signals encountered in real data.

## Methods

In this section we describe our algorithm for automated, whole spectrum scale Gaussian mixture modeling (GMM) of proteomic mass spectra and for MS peak detection based on Gaussian mixture representation. We first introduce the notations for such spectra and their Gaussian mixture models. Afterwards, we first present the main idea of the algorithm.

A typical proteomic mass spectrum contains data on mass-to-charge (m/z) values of the registered ions, denoted by *x*_*n*_ versus their abundances i.e., the numbers of counts from the ion detector, denoted by *y*_*n*_, *n* = *1,2,…,N*. The number of data points in the spectrum is denoted by *N*. In real experiments the analyzed datasets most often consist of more than one spectrum, multiple counts by *y*_*mn*_, *m* = *1,2,…,M* correspond to each point *x*_*n*_ along the m/z axis, where *m* denotes the index of the spectrum and *M* is the number of the spectra.

As the model for proteomic mass spectra, we use the univariate Gaussian mixture probability density function of the form

$$f(x_{n})=\sum_{k=1}^{K}\alpha_{k}f_{k}(x_{n},\mu_{k},\sigma_{k})$$ (1)

where *K* is the number of Gaussian components, *α*_*k*_, *k* = 1,2,…*K* are component weights (mixing proportions), which sum up to 1,

$$\sum_{k=1}^{K}\alpha_{k}=1$$ (2)

and *f*_*k*_ (*X*_*n*_, *μ*_k,_ *σ*_*k*_)denotes the probability density function of the Gaussian distribution.

$$f_{k}\left(x_{n},\mu_{k},\sigma_{k}\right)=\frac{1}{\sigma_{k}\sqrt{2\pi }}e^{-\frac{{\left(x_{n}-\mu_{k}\right)}^{2}}{2\sigma_{k}^{2}}}$$ (3)

In Eqs (1) and (3) *μ*_*k*_ and *σ*_*k*_, *k* = *1,2,…K*, are means and standard deviations of the Gaussian components, respectively.

### Scaling

The mixture model (1) must be appropriately scaled. Due to finite sensitivity of the ion detector, numbers of counts in the average spectrum, *y*_*n*_, correspond to ranges (intervals) in the m/z axis, (x_n_ − Δ_n_ / 2, x_n_ + Δ_n_ / 2), where Δ_n_ is the width of the interval centered at *x*_*n*_. In other words the data are binned and the numbers of counts *y*_*n*_ are modeled by the multinomial probability distribution with probabilities given by areas of bins [28]. For real proteomic MS data bin widths Δ_n_ are changing with *n*; they are narrower for low *x*_*n*_ and wider for high *x*_*n*_. One can assume that bins are dense, i.e., their areas are well approximated by products of bin widths and values of the probability density functions at bin centers, which corresponds to the following model

$$y_{n}=\eta \Delta_{n}\sum_{k=1}^{K}\alpha_{k}f_{k}(x_{n},\mu_{k},\sigma_{k})$$ (4)

The parameter *η* in (4) is called the total ion current (TIC) [29]. From (4) the value of the total ion current *η* is

$$\eta =\sum_{n=1}^{N}y_{n}$$ (5)

We call model (4) the globally scaled model of the MS signal *y*_*n*_ (due to its function of changing the scaling from probability densities to ion counts).

We are, however, more interested in using locally scaled models for the MS signal *y*_*n*_. When we analyze only a small fragment of the spectral signal *n*_min_ ≤ *n ≤ n*_max_, we can assume constant bin widths Δ_n_ = Δ. This allows for writing the locally scaled model in the following form

$$y_{n}=\sum_{k=1}^{K}w_{k}f_{k}(x_{n},\mu_{k},\sigma_{k}),n_{\text{min}}\leq n\leq n_{\text{max}}$$ (6)

Where *w*_*k*_ = *sα*_*k*_, (*s* = *η*Δ),. The scale factor *s* can be computed “locally”.

$$s=\frac{\sum_{n=n_{min}}^{n_{max}}y_{n}}{\sum_{n=n_{min}}^{n_{max}}\sum_{k=1}^{K}\alpha_{k}f_{k}(x_{n},\mu_{k},\sigma_{k})}$$ (7)

### Main idea of the algorithm

Fitting the scaled mixture model (4) or (6) to spectral data *x*_n_, *y*_*n*_, is done by using expectation maximization (EM) algorithm [28, 30]. A special version of the EM algorithm for binned data [28] is needed, described in detail in S1 File.

Fitting a mixture model to MS data by EM iterations at the whole spectrum scale is, however, impractical (impossible) due to reasons described in detail at the beginning of the “Discussion and Conclusion” section. Therefore we have developed a method to decompose the MS signal into smaller fragments. Our method uses the property of the MS signal that after removing baseline (which is a wide component of the spectral signal) the remaining components are relatively narrow.

**Fig 1:**
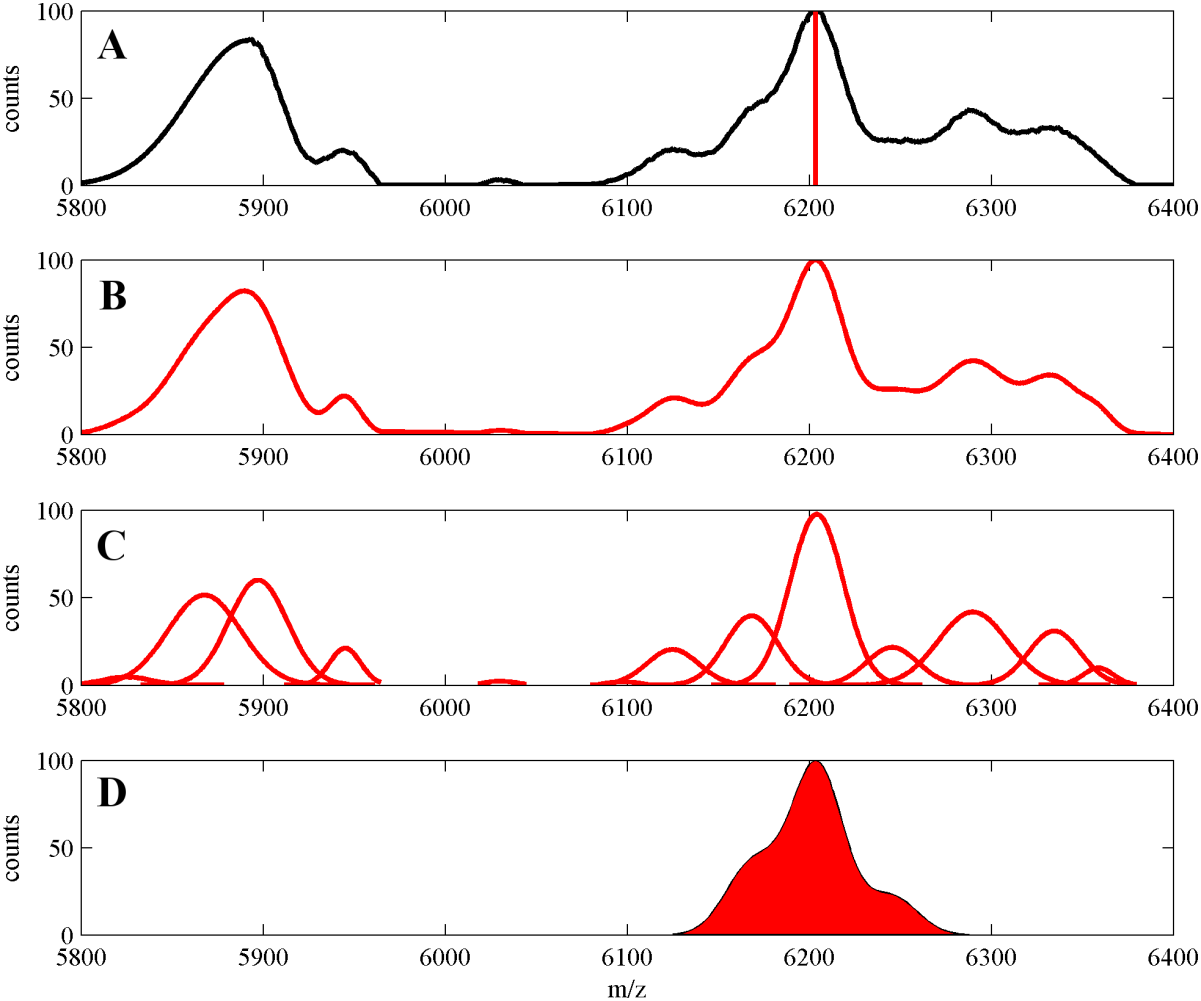
**(A)** Splitter segment—a fragment of the MS signal (black) around the clear peak detected in the MS signal (red vertical line close to m/z = 6200). **(B)** GMM of the splitter-segment signal, splitter-segment signal (black). **(C)** Components of the Gaussian mixture model (red). **(D)** Splitter computed on the basis of the clear peak (filled red).

**Fig 2:**
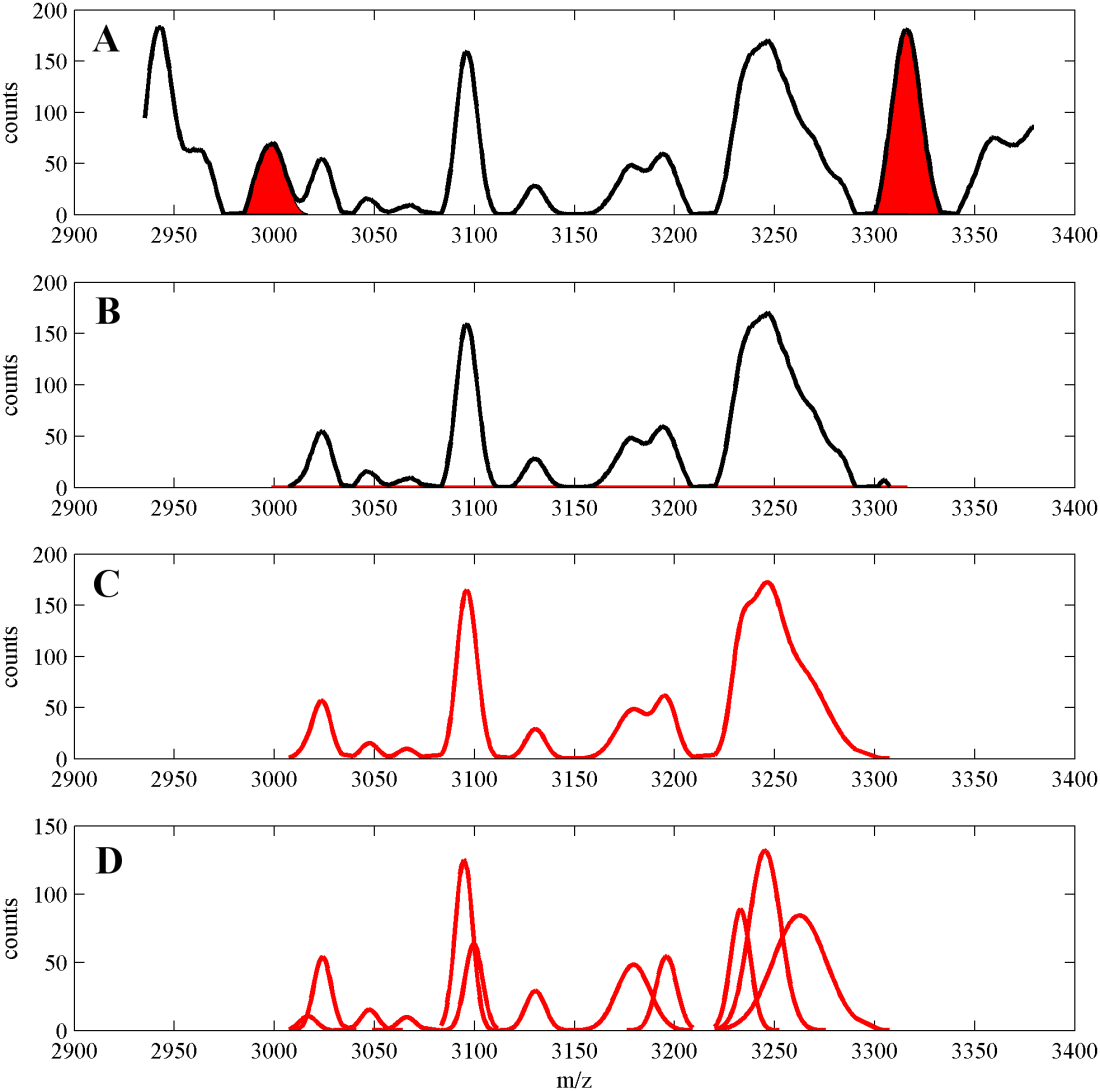
**(A)** Fragment of the MS signal (black line) with two neighboring splitters (filled red). **(B)** Segment (black line) resulting from subtracting the splitters signals from the MS signal. **(C)** Gaussian mixture decomposition of the segment signal (red) **(D)** Components of the Gaussian mixture model (red).

The main idea of the algorithm is partitioning the mass spectrum. The partitioning algorithm uses the following concepts: “splitters”, “clear peaks”, “splitter segments” and “segments”. These concepts are defined below and further explained and illustrated (Figs 1 and 2) in the remaining part of this subsection. Figs 1 and 2 are plotted for one artificially generated MS datasets available in S2 File.

### Definitions

A **clear peak** is one of the peaks of the mass spectrum, chosen on the basis of its quality index and its position in the spectrum. We also use another term, **splitting peak**—synonymously to the term clear peak.

A **splitter** is a fragment of the mixture model of a protein mass spectrum, which contains a clear peak.

A **splitter—segment** is a suitably chosen fragment of the mass spectrum, which contains a clear peak (around a clear peak).

A **segment** is a fragment of the mass spectrum obtained by subtracting two neighboring splitter signals from the mass spectrum signal.

### Explanations

An example of a clear peak and the related splitter in the protein mass spectrum is shown in Fig 1. The position of the clear peak is marked by the vertical red line in the upper plot (A) and the splitter signal is filled in red in the lower plot (D).

In order to compute (estimate) the splitter signal we “cut out” a fragment of the MS signal around the clear peak (Fig 1A). This fragment of the MS signal is a splitter—segment. We perform Gaussian mixture decomposition of the splitter—segment (Fig 1B). Cutting (truncating) the MS signal leads to possible errors in modeling. However, on the basis of our assumption of narrow components we expect that errors occur only close to boundaries of the splitter-segment and that they do not affect the model of the splitter (in the middle). Intuitively, in the vicinity of the clear peak the MS signal can be modeled by either only one or a small number of Gaussian components. These components are reliable parts of the decomposition of the MS signal into mixture of Gaussians.

For a given MS signal we need a set of splitters. Therefore, in the phase 1 of our algorithm, for a given MS signal, we search for a set of splitters by applying a heuristic procedure, which uses a peak detection algorithm as its first step. In principle any peak detection algorithm can be applied. In our implementation we used “mspeaks” function from Matlab Bioinformatics toolbox [31] (with the default options). The heuristic procedure for searching for clear peaks (splitting peaks) is designed such that it returns a set of clear peaks, which are neither too close nor too far from each other and each of them is of sufficient quality (measured by the ratio of the peak height and heights of the neighboring lowest points of the MS signal). Then, by using EM iterations we compute decompositions of splitter-segments (fragments of MS signals around each of the splitting peaks), as shown in Fig 1, and we obtain models of all splitters signals.

Since models of splitters signals are reliable parts of Gaussian mixture decomposition of the MS signal, in the phase 2 of our algorithm we subtract splitters signals from the MS signal, which leads to splitting (partitioning) the whole spectrum into separate fragments—segments. Then, segments are decomposed into Gaussian mixtures, again by using EM iterations. The idea of the phase 2 of our algorithm is illustrated in Fig 2. In Fig 2A we present a fragment of the MS spectrum with two (neighboring) splitters. In Fig 2B we show the MS signal of the segment obtained by subtracting splitters models signals from the MS signal. In Fig 2C and 2D we show Gaussian mixture decomposition of the segment signal from the middle plot (C—GMM and D—GMM components).

Finally, we aggregate all the computed GMM components into one set, which is a whole-spectrum mixture model of the MS signal. A more detailed description of the steps of our algorithm is given in S1 File. A Matlab implementation of our algorithm and exemplary data are available as S2 File.

## Results

In this section we present some evaluations of the performance of our algorithm and comparisons to methods of analyses of MS signals based on picking spectral peaks. The presented results concern both simulated (low resolution) datasets and real proteomic datasets of low and high resolution.

Computations for all datasets were performed with the use of the computational server with two hi-end Intel Xeon X5680 processors (3.4 GHz in normal work, 3.6 GHz in turbo mode) and 32 GB DDR3 1333 Mhz RAM memory. Average processing times were, for one low resolution spectrum (approximately 10000 m/z points and 100–500 components)– 1.5 minutes and for one high resolution spectrum (approximately 100000 m/z points and 1000–15000 components)– 25 minutes.

### Simulated data

First we applied our algorithm as a tool for peak detection for simulated proteomic MS spectra. We compare our algorithm to two existing procedures for protein MS peak detection published in [2] and [3]. Our choice of the reference algorithms is based on the comparative studies [32–34] of algorithms for peak detection for the MS. The algorithm and associated computer program (R environment) published by Du and coauthors [3] was rated high in all comparisons studies as showing high sensitivity for peak detection with quite low false discovery rate. It is based on computing continuous wavelet transform (CWT) of the spectral signal, with the “Mexican Hat” mother wavelet function, and relating spectral peaks to the “ridge” lines in the parameter space. The algorithm developed by Coombes and coauthors in [2] (with publicly available implementation in the Matlab environment) was rated lower in comparisons [32–34]. When using this algorithm it is quite difficult to compromise between sensitivity of peak detection and false discovery rate. However, its advantage is that it uses natural ideas for peak detection, smoothing (with the use of wavelet functions) and differentiation of smoothed spectral signal. For the three compared algorithms we use the following abbreviations: MS-GMM—for our algorithm, CWT (continuous wavelet transform)–for the algorithm from [3] and CROM (Cromwell)–for the algorithm from [2].

Similarly to other studies devoted to comparisons of peak detection algorithms, [32–34], we use mass spectra, obtained with the use of the virtual mass spectrometer (VMS), [35], where the true positions of peaks in spectrum are known. We additionally change the structure of the simulated data by assuming different numbers of true peaks in the spectra, and we study their influence on the detection power of different algorithms.

### Spectral peaks

Three algorithms MS-GMM, CWT and CROM are compared with respect to their efficiency in detection of spectral peaks. Spectral peaks are features of mass spectra given by lists of m/z values. Peak detection algorithms are designed such that estimated spectral peaks should be as close as possible to the true m/z values corresponding to peptides (proteins) in the analyzed samples. In the case of CWT and CROM algorithms, spectral peaks m/z values are estimated by maxima detection procedures accompanied by noise rejection and smoothing. In the case of MS-GMM spectral peaks are given by m/z values corresponding to expectations of Gaussian components in the GMM. These Gaussian components are obtained in the post-processing step of the algorithm described in detail in S1 File.

### Virtual mass spectrometer datasets

Synthetic spectral datasets are obtained with the use of the VMS algorithm/tool [35] based on the physical principles underlying mass spectrometry instruments. This tool enables the generation of realistic virtual spectra with known underlying protein (peptide) compositions, and has already been widely used by many authors, [32, 34]. VMS signal contains the same parts as those (hypothetically) encountered in actual spectral signals, namely the true spectral signal consisting of a sum of overlapping Gaussian components (each corresponding to a protein or peptide species) multiplied by a random multiplicative factor adjusting for random amounts of proteins/peptides ionized and desorbed from each slide, a baseline signal and a zero mean Gaussian error with the variance given by a smooth function of m/z. For a given protein/peptide ion (i.e. spectral component) we summarize its distribution across samples by three quantities: its prevalence defined by the proportion of samples in the population containing the component, the mean and the standard deviation of corresponding peak intensity across samples that contain the component.

By using the VMS algorithm we have generated five datasets with different true numbers of protein (peptide) species, 100, 150, 200, 250 and 300. The detailed description of the scenario for simulating artificial (synthetic) datasets is presented in S1 File, and the generated spectra are provided in S3 File.

### Comparisons of performances of algorithms

We compute several performance indexes, useful to characterize/compare results obtained by different algorithms. The specificity index (defined by false discovery rate) is abbreviated by FDR. FDR is the number of peaks among those detected by the procedure which do not correspond to the true peaks, divided by the number of all peaks detected by the procedure. The sensitivity index is abbreviated by S. S is the number of true peaks detected by the procedure divided by the number of all true peaks in the sample. We also aggregate the performance measures FDR and S into one index, F1 (defined as the harmonic mean of 1-FDR and S)

$$F1=\frac{2\left(1-FDR\right)S}{1-FDR+S}$$ (8)

Obviously, higher values of F1 index imply better performance and lower values—poorer performance of the evaluated method. Finally, we also report the number of peaks detected by a peak detection algorithm.

**Fig 3:**
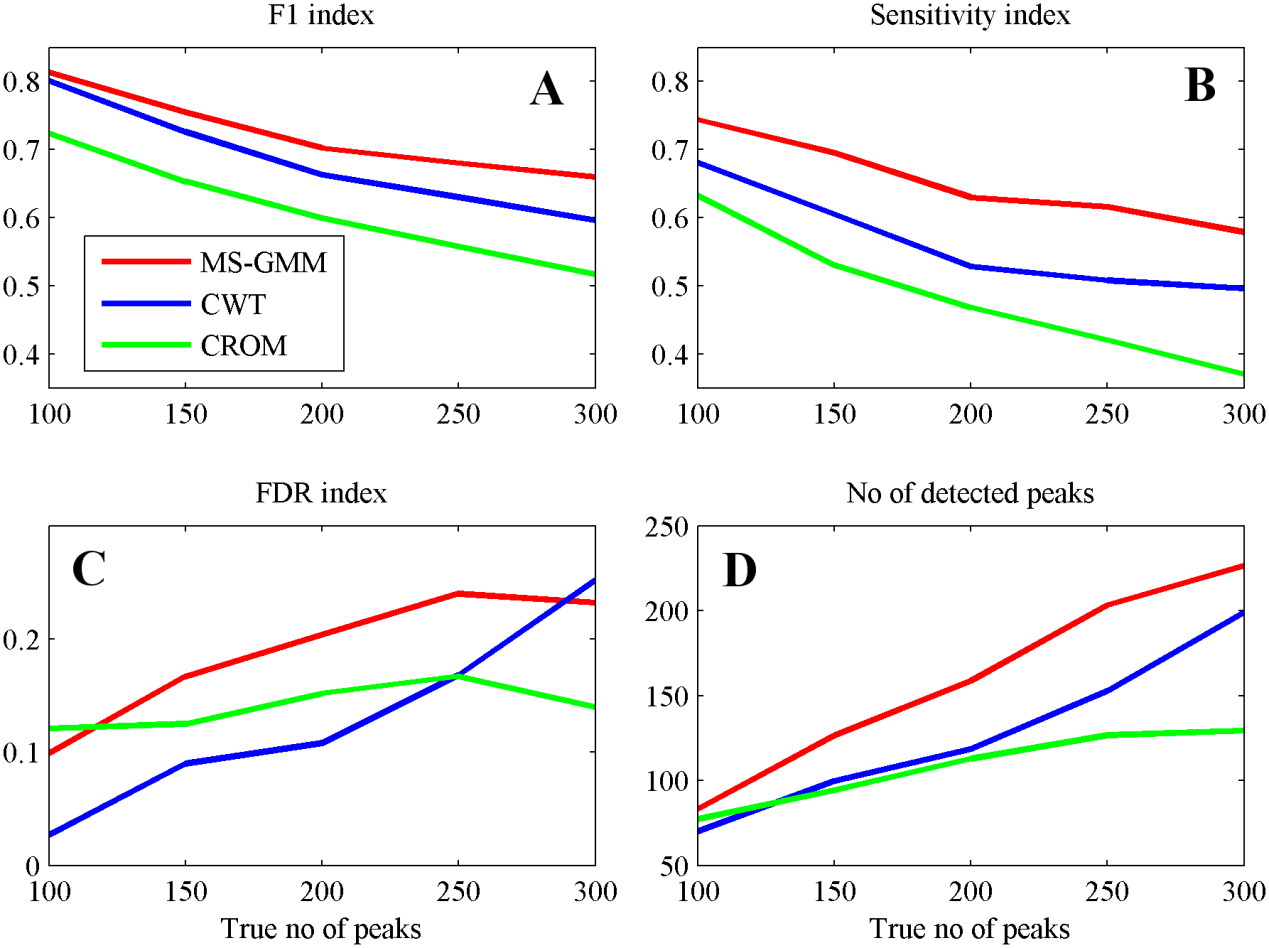
Performance indexes for the three peak detection algorithms applied for mean spectra in the simulated datasets. **(A)** F1 score. **(B)** Sensitivity. **(C)** FDR. **(D)** No of detected peaks. Colors: MS-GMM—red, CWT—blue, CROM—green.

Results of comparisons are presented in Fig 3 where we show plots of indexes F1, FDR, S and the numbers of (hypothetical) peaks detected by the algorithms versus numbers of true peaks in the simulated spectra.

Performance indexes for all algorithms, MS-GMM, CWT and CROM show similar patterns of change. As reported in published literature, CROM achieves the lowest values of the F1 index for the whole range of true numbers of peaks in virtual spectra. Differences between algorithms are also seen when comparing numbers of the detected (hypothetical) peaks, shown in the Fig 3D. All algorithms underestimate the number of peaks.

Our algorithm MS-GMM exhibits the best performance in terms of values of the F1 index, for the whole range of values of true numbers of peaks in the virtual spectra. The possibility of tuning the parameter of this algorithm to achieve best compromise between sensitivity and FDR follows from highest values of sensitivities of our algorithm compared to other algorithms (Fig 3B). Our algorithm is also closest to the truth when estimation the number of peaks in the spectral signal is considered.

In Table A in S1 File, optimal values of parameters used in algorithms MS-GMM, CWT and CROM, when computing performance indexes. We also show, in Fig C in S1 File, ROC curves (FDR versus sensitivity) obtained by applying the algorithms with different values of their adjustable parameters. These curves demonstrate the increase of the sensitivity, at the same values of FDR, obtained by our MS-GMM algorithm, when compared to CWT and CROM.

**Fig 4:**
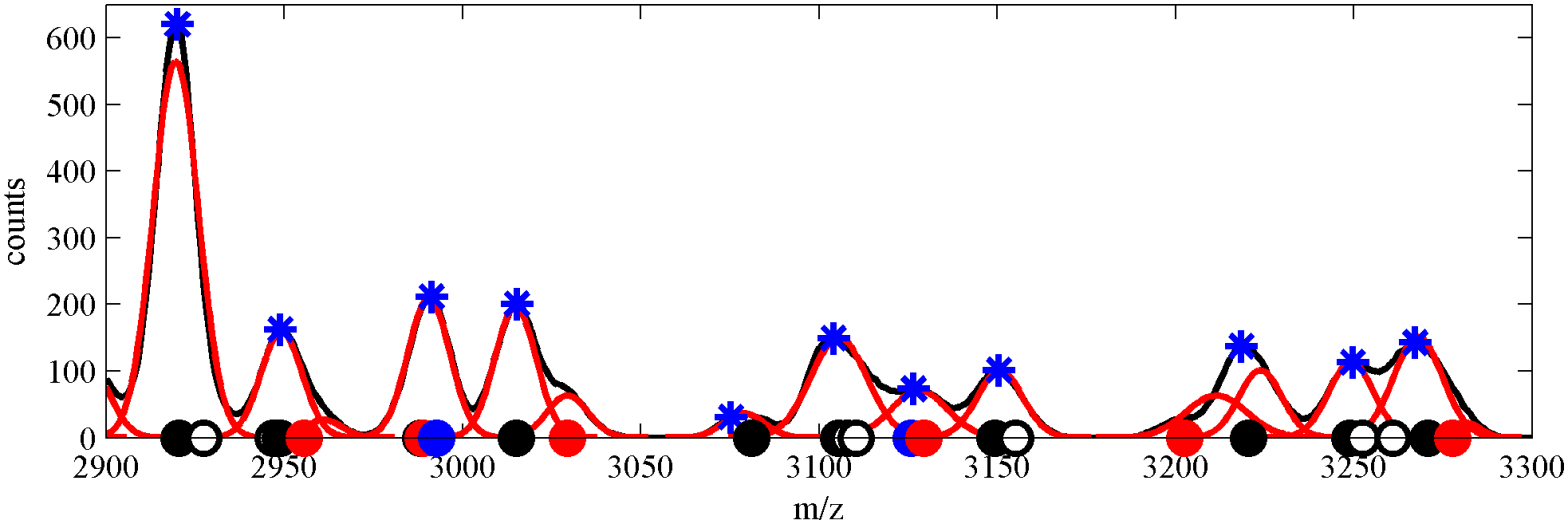
Fragment of one virtual MS dataset (with 200 peaks, m/z range 2900–3300 Da). Comparison of MS-GMM and CWT. MS signal (black), GMM model components (red), peaks detected by CWT algorithm (blue asterisks). Positions of true peaks in the spectral signal are marked by circles symbols and detection status is depicted by colors: peak detected only by MS-GMM method (red), peak detected only by CWT method (blue), peak detected by both MS-GMM and CWT (black), peak not detected by any of algorithms (empty circle).

Improvement of performance (increased sensitivity) of peak detection achieved by using our Gaussian mixture model is obtained thanks to detection of “hidden” peaks in the spectral signals. This is additionally illustrated in Fig 4 below, where we have reproduced a small fragment (2900–3300 Da) of one spectral signal (with 200 true peaks). The plot includes positions of peaks detected by using CWT algorithm (blue asterisks) and components (peaks) detected by using our algorithm MS-GMM (red Gaussian curves). Along the m/z line (x-axis) we have marked with circles all true peaks in the spectrum (within the analyzed range) and we have additionally colored the circles depending on the detection status using the following code: detected only by MS-GMM method—red, detected only by CWT method—blue, detected by both MS-GMM and CWT—black and not detected by any of algorithms—empty circle. One can see several examples of hidden peaks, which have been detected thanks to the use of the Gaussian mixture model.

### Real proteomic datasets

We show some results of application of our GMM decomposition approach and comparisons to peak picking algorithms for real proteomic datasets.

### Aurum dataset

The first real dataset, which we have analyzed in this paper is the Aurum Dataset [27]. The Aurum dataset contains high resolution MS and MS/MS spectra of 246 human proteins of known amino-acid sequences expressed in Escherichia coli, individually purified and trypsin-digested. MS and MSMS spectra were registered with the use of the ABI 4700 MALDI TOF/TOF mass spectrometer.

We have analyzed MS data in the Aurum dataset. The set of the MS data in the Aurum dataset includes six series of experiments (batches) of registering MS, each containing 192 spectra, which in total gives 1152 spectra. Each of the spectra in the Aurum dataset is accompanied by a list of ground truth, m/z values corresponding to peptide species present in the registered samples. We have computed GMM decompositions and peak detection by CWT algorithm for all 1152 spectra and we have performed a comparison between these two approaches.

For the Aurum spectra we highlight improvement of accuracy of estimation of positions of peaks achieved by application of MS-GMM. This improvement is related to skewness of shapes of components of spectra (spectral peaks) corresponding to the peptide species and concerns accuracy of estimation of positions of peaks. For the skewed components of MS the position of the maximum of the MS signal and the true m/z of the peptide species may not coincide [19]. According to the methodology outlined by Kempka and coauthors [19] (see also references therein), skewed spectral peaks can be modeled by a mixture of two Gaussian components and the position of the higher-narrower component is a better estimate of the m/z value of the peptide species than the maximum point of the peak.

We have performed computations analogous to those described in [19] for all 1152 Aurum MS spectra. We have tuned both MS-GMM and CWT algorithms for high sensitivity (in order to detect all ground truth peaks) and we have compared relative absolute errors of estimates of the m/z values between GMM and CWT algorithms. Absolute relative error is defined as

$$RE=\frac{\left|m/z_{ESTIMATED}-m/z_{TRUE}\right|}{m/z_{TRUE}}$$ (9)

When we have modeled the spectrum by the GMM method, the estimate of m/z was the position of the higher-narrower Gaussian component closest to the true m/z, and for the CWT method the estimate of the m/z value was the position of the detected peak closest to the true m/z.

**Fig 5:**
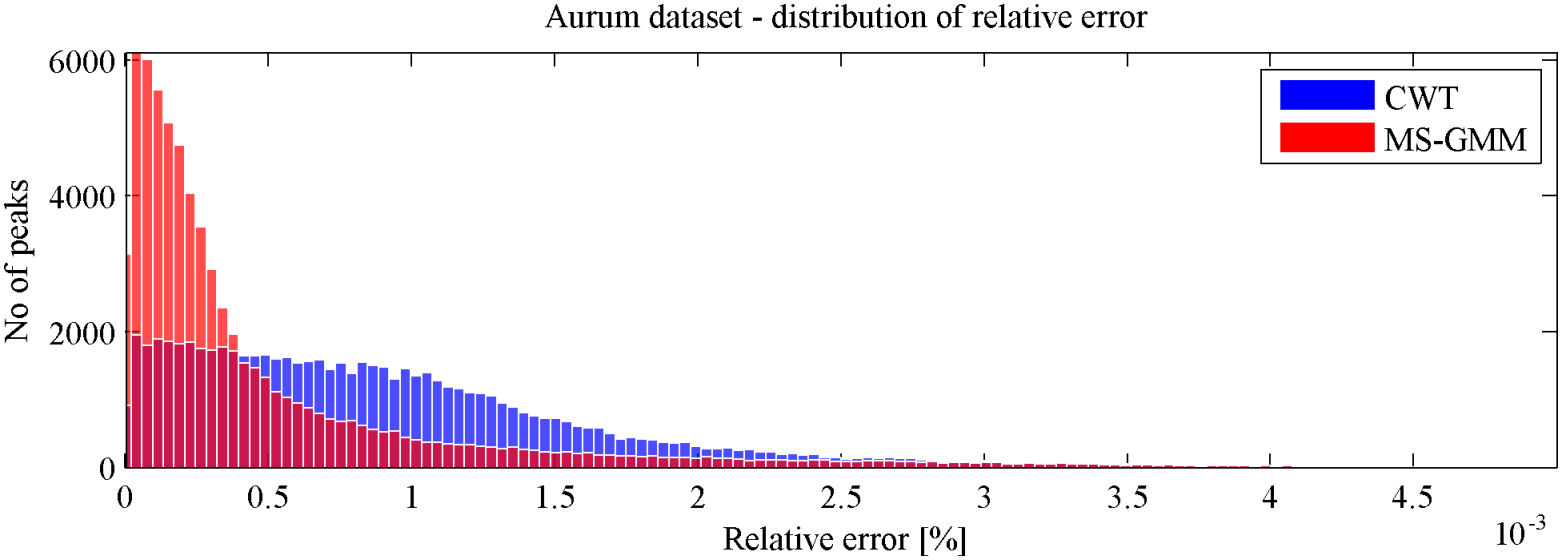
Aurum dataset—distributions of absolute relative errors in estimating positions of peaks, represented by histograms, for MS-GMM (red) and CWT (blue) algorithms.

Results of comparison of relative absolute errors between the two algorithms are shown in Fig 5. Histograms of absolute relative errors corresponding to MS-GMM (red) and CWT (blue) are presented. One can see that MS-GMM algorithm outperforms the CWT algorithm in the aspect of the value of the absolute relative error (9). Values of RE obtained with the use of MS-GMM are on the average closer to zero than values obtained by using the CWT algorithm.

Distributions of absolute relative errors shown in Fig 5 are right-skewed. Therefore it is statistically sound to report medians and interquartile ranges for comparisons between these distributions. In Table 1 we present median values and interquartile ranges (IQR) of distributions of the absolute relative errors, obtained by using either MS-GMM or CWT algorithms, for all six series of experiments (batches) in the Aurum dataset.

**Table 1:** Median values and IQRs of distributions of RE, resulting from MS-GMM or CWT algorithms, for all batches in the Aurum dataset [27].

	MS-GMM		CWT	
	Median [%]	IQR [%]	Median [%]	IQR [%]
Batch T10467	0.198E-03	0.333E-03	0.664E-03	0.824E-03
Batch T10622	0.259E-03	0.536E-03	0.810E-03	0.969E-03
Batch T10645	0.246E-03	0.451E-03	0.818E-03	0.928E-03
Batch T10707	0.296E-03	0.653E-03	0.762E-03	0.887E-03
Batch T10739	0.294E-03	0.605E-03	0.861E-03	1.015E-03
Batch T10761	0.302E-03	0.677E-03	0.879E-03	1.044E-03

**Fig 6:**
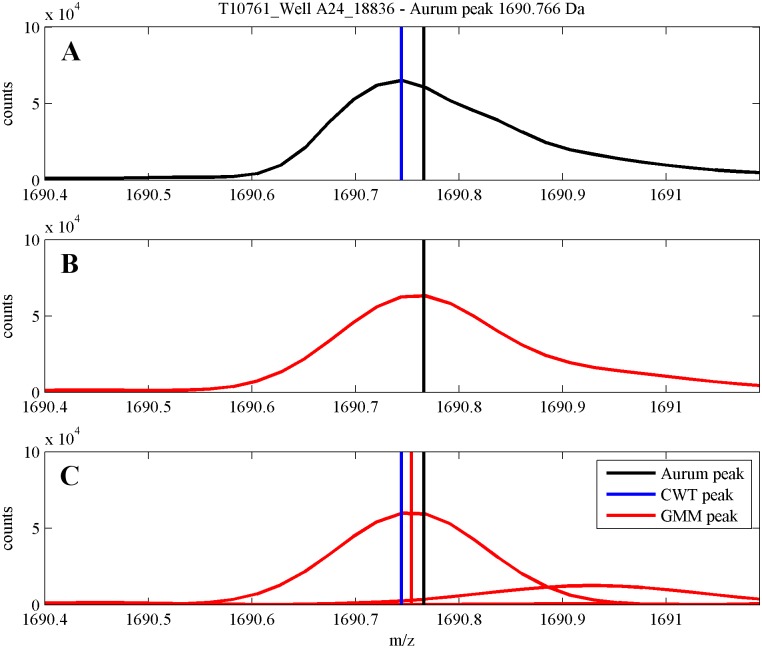
Short fragment of MS including one ground truth Aurum peak m/z = 1690.766 Da from the spectrum T10761_Well A24_18836 and its GMM. **(A)** MS fragment, **(B)** GMM decomposition, **(C)** GMM components. We additionally mark, by vertical lines m/z positions, black: true Aurum peak 1690.766, red: m/z estimate by using MS-GMM, blue: m/z estimate by using CWT algorithm.

In Fig 6 we present a short fragment of MS T10761_Well A24_18836 from the Aurum dataset including one ground truth peak m/z = 1690.766 Da from the spectrum. We present the MS fragment, its GMM decomposition and GMM components. We also mark, by vertical lines ground truth position of the peak (1690.766 Da) and m/z positions corresponding to its estimates by CWT and MS-GMM algorithms. One can see that the shape of the peak is right-skewed and that the MS-GMM estimate of peak’s m/z is closer to the true value of m/z than the CWT estimate.

### Other proteomic datasets

In the majority of cases of analyses of real datasets, true compositions of samples are not known, which makes impossible comparisons of different algorithms based on differences between detected and true positions of peaks. For such real proteomic spectral datasets comparisons of GMM modeling to methods based on spectral features defined by peaks can still be done on the basis of indirect methods, e.g., on the basis of comparing efficiencies of spectral classifiers using different definitions of spectral features. We are, however, deferring such analyses to separate studies. Here, instead, we provide some technical comments on results of analyses of two real proteomic datasets (low resolution dataset and high resolution dataset) concerning abilities of GMM modeling method to detect certain spectral components and concerning shapes of spectral signals encountered in real data. Importance of these properties of GMM modeling was highlighted in the presented earlier analyses of the simulated datasets and the Aurum dataset.

### Low resolution dataset

The low resolution dataset comes from published clinical study aimed at detection of colorectal cancer using serum peptidome profiling by MALDI-ToF mass spectrometry [25]. The dataset included 116 MALDI-ToF spectra of the low-molecular-weight fraction of serum proteome of cancer patients and healthy volunteers, each covering the m/z range 960–11,169 Da. Spectra were registered by Ultraflex MALDI-ToF spectrometer (Bruker Daltonics) working in the linear mode. The raw spectra contained approximately 45000 points along m/z axis. We used operations of averaging and binning described in the original paper, which resulted in “low resolution” spectra including approximately 10000 data points along m/z axis (~1 point per Da). Using our algorithm with the default settings resulted in computing the GMM model with 472 components.

**Fig 7:**
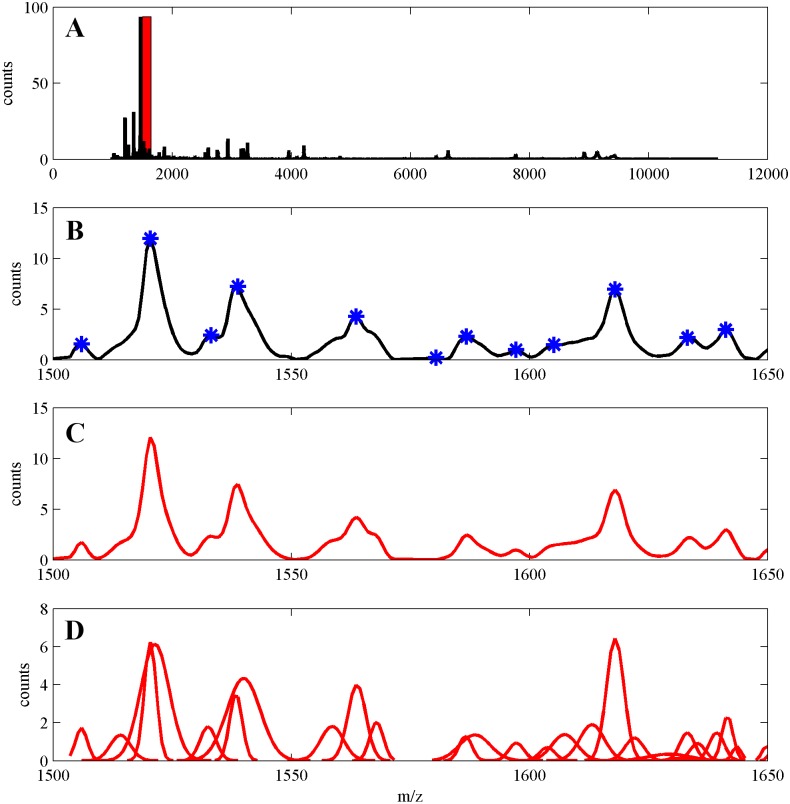
**(A)** Whole average MS corresponding to the serum peptidome for the data in [25]. Small fragment of this MS, within the range 1500–1650 Da is highlighted by a red rectangle. **(B)** Zoomed out fragment 1500–1650 Da of the MS. Peaks detected by using the CWT algorithm are marked by blue asterisks, **(C)** GMM signal of the fragment (red), **(D)** GMM components (red).

In Fig 7 we illustrate results of this computational experiment for the case where no operations of post-processing of the GMM model were applied. In the upper plot (A) whole average MS is shown. It is seen that due to large size of MS it is not possible to recognize any details of the structure of MS. Therefore in (B) we show a short fragment of MS from (A). Along with the fragment of MS in (B) we also show, with blue asterisks, positions of peaks detected by using the CWT algorithm. In (C) we present its GMM and in (D) GMM components. From the plots in Fig 7 one can again see several examples of parts of spectral signal, modeled by Gaussian components, not detected by peak detection algorithm (hidden peaks). In the right-hand part of the spectrum model one can see a low, wide component, which seems to result from some residue of baseline. It is seen that due to the fact that no post-processing was applied, there is some excess of Gaussian components used to modeling.

Application of the post-processing algorithm for MS-GMM (with parameters optimized for the case of 300 true peaks in the spectrum, see Table A in S1 File) reduced the number of GMM components from 472 to 391. The total number of peaks detected by using the CWT algorithm was 258. Parameters of the CWT algorithm were also optimized for the case of 300 true peaks in the spectrum—see Table A in S1 File. Application of the detection threshold 0.3% leads to the estimate of overlap between MS-GMM and CWT estimates of peaks positions including 141 elements. Differences between two sets of peaks follow from presence of hidden components, such as those seen in (Fig 7B and 7D).

### High resolution dataset

**Fig 8:**
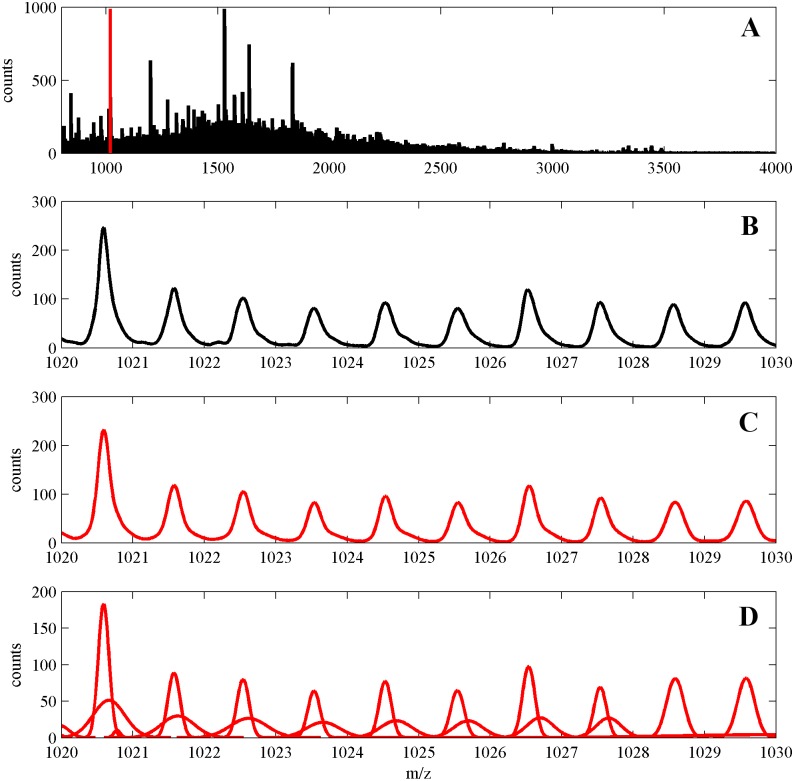
**(A)** High resolution mean spectrum corresponding to our own proteomic dataset of head and neck cancer tissues, small fragment (1019–1030 Da) is marked by a vertical red line. **(B)** Zoomed out fragment 1019–1030 Da. **(C)** GMM. **(D)** GMM components.

The high resolution MS dataset was generated in our team during characterization of head and neck cancer tissue proteome [26]. In the study a post-operative tissue sample was analyzed using MALDI Imaging Mass Spectrometry (MALDI-IMS). Tissue section processed with trypsin digestion was imaged with 50–100 μm raster using UltrafleXtreme MALDI-ToF spectrometer (Bruker Daltonics) working in the reflectron mode. Spectra were registered in the 800–4,000 Da range, which resulted in 20000 spectral signals, each containing 100000 data points along the m/z axis (i.e., ~30 points per Da, which could be considered as “high resolution” spectra). We have computed a mean spectrum (over 20000 signals) and we have decomposed it according to the GMM model, using our algorithm with the default settings, which resulted in obtaining in total 6216 components. In Fig 8 we illustrate results of computations. In the upper plot (A) we plot whole mean MS. Again due to large size of MS one cannot recognize details of the structure of MS. Therefore in (B) we zoom out a short fragment (1019–1030 Da) of mean spectrum. In (C) the GMM is shown and in (D) we presented GMM components. One can see a characteristic high resolution MS signal isotopic pattern with neighboring peaks occurring in the distance 1 Da. One can also observe that isotopic parts of the spectral signal are right skewed. Application of our algorithm results in modeling each of them by two Gaussian components, analogously to the case of Aurum spectra.

## Discussion and Conclusion

The standard approach to modeling signals by mixtures of Gaussian component functions is by using expectation-maximization (EM) algorithm—a recursive procedure for maximization of the log likelihood function [28, 30]. However, there are serious obstacles on the way to developing an EM recursive algorithm for fitting a mixture model to the multi-component proteomic mass spectra. The first obstacle stems from the difficulty in setting initial conditions for EM iterations. Fitting a mixture model with a large number of components to data is difficult due to problems with setting appropriate initial conditions for the EM algorithm. The problem of setting the initial values of mixture parameters for the EM algorithm has been researched numerous times in the literature [36–39]. However, the published approaches are practical only for mixtures with a relatively low number of components. When the number of components increases, the precision of estimation of mixture parameters obtained with the use of the mentioned methods of initialization rapidly decreases. This makes the published methods inapplicable for mixtures with hundreds or even thousands of components encountered in spectra registered for complex proteomic mixtures (like serum or cancer tissue). An approach useful for setting initial components for the EM iterations dedicated to such spectra was proposed in [18]. This approach applies an algorithm for detecting MS peaks as a first step and then sets initial mean values of components equal to detected locations of peaks. While the idea of using available information on locations of peaks of the spectrum is certainly reasonable and useful, the proposed approach still suffers from serious drawbacks: (i) EM iterations started with mean values of components positioned at MS peaks can still converge to undesired solutions due to imprecisions of initial values of component weights and standard deviations, (ii) the method is blind to hidden components, which are not identified (detected) by MS peaks, (iii) the method may require launching EM iterations at the full spectrum scale, which can be difficult (impossible) for large datasets. The second obstacle is the size of the proteomic MS data. For very large datasets, with numbers of points along the m/z axis of a spectrum of the order of tens or even hundreds of thousands, executing (iterating) EM algorithm can be difficult due to large sizes of the necessary data structures and problems with the slow convergence.

We have managed to overcome the previously encountered difficulties and to develop an efficient method for the automated whole spectrum decomposition of MS signals into Gaussian mixtures. The idea of the algorithm is based on partition of the spectral signal into separate fragments. The partition is obtained by defining “splitters” (fragments of the GMM model, which contain “clear peaks”, as shown in Figs 1 and 2). The possibility of the partition by splitters follows from properties of MS signals (with removed baselines). In the baseline corrected MS signals components are relatively narrow, which excludes long ranging overlaps. Separate segments are decomposed into GMM model by using EM iterations initialized with the use of the high efficiency algorithm. Despite the multi—step design of our algorithm its ideas are simple. Partitioning of spectra allows for separate analyses leading to mixture models of sufficient precision. Aggregating results of decompositions of segments leads to Gaussian mixture model of the whole spectrum.

Separate decompositions of MS segments allow for estimation of whole spectrum Gaussian mixture models of MS signals of arbitrarily large sizes (proven by automated analyses of high resolution spectra with numbers of m/z values of orders of hundreds of thousands). Separation also enables easy parallelizing of computations, which can be used to elaborate high efficiency computational environments based on multi-processor hardware systems. Efficiency of the idea of partitioning can be exemplified by comparing partitioned and un-partitioned versions of GMM decomposition algorithms Partitioned version of the GMM decomposition algorithm allows for obtaining results in a shorter time and leads to mixture model of a better quality, when compared to un-partitioned version of the algorithm. Examples of such comparisons are shown in S1 File.

When using the obtained mixture models for peak detection we have encountered a problem of selecting peaks from the set of mixture components. This problem arises because some of the mixture components obtained in the iterative EM algorithm may not correspond to spectral peaks. We have proposed a solution to this problem by a post-processing algorithm described in S1 File, including a threshold value for component weights and a method for merging Gaussian components with a tunable parameter MZ-thr. We have compared the peak detection method based on our GMM decomposition algorithm to two literature algorithms for peak detection (CWT, CROM), on the basis of artificially generated MS signals, and we have demonstrated its supremacy (Fig 3 and Fig C in S1 File).

For the publicly available Aurum dataset [27] we have highlighted the supremacy of the GMM approach over the pick picking algorithms when comparing relative accuracies of estimating positions of MS peaks between MS-GMM and CWT algorithms. Consistent to findings in [19], application of MS-GMM leads to estimates of m/z values of peaks of lower relative error than estimates obtained by using CWT algorithm.

Apart from improvements of the efficiency of peak detection/estimation demonstrated in this paper, there are also other areas of possible applications for an algorithm for the automated, whole spectrum scale GMM decomposition of MS signal. Gaussian mixture modeling of MS signals can be potentially used as a tool for smoothing and de-noising spectral signals, for modeling and/or removing baselines in the spectra, for MS signals peak quantification, for MS signal compression and for spectral deisotoping algorithms. Other applications can involve using mixture models for defining spectral features to be further used in construction of protein spectral classifiers.

## Supporting Information

S1 File Supplementary Materials. (DOCX)

S2 File A Matlab implementation of the proposed algorithm and exemplary data. (ZIP)

S3 File Simulated data with true peaks. (ZIP)
