# Supplementary material for: Signal Partitioning Algorithm for Highly Efficient Gaussian Mixture Modeling in Mass Spectrometry
Source: PLoS One. 2015 Jul 31;10(7):e0134256. doi: 10.1371/journal.pone.0134256 (PMC4521892; doi:10.1371/journal.pone.0134256)
Supplement: S1 File — (DOCX) [file pone.0134256.s001.docx]

**SUPPLEMENTARY MATERIALS**

**Signal Partitioning Algorithm for Highly Efficient Gaussian Mixture Modeling in Mass Spectrometry**

Andrzej Polanski ^1^, Michal Marczyk^2^, Monika Pietrowska^3^, Piotr Widlak^3^, Joanna Polanska^2*^

^1^Institute of Informatics, Silesian University of Technology, Gliwice, Poland

^2^Institute of Automatic Control, Silesian University of Technology, Gliwice, Poland

^3^Maria Sklodowska-Curie Memorial Cancer Center and Institute of Oncology, Gliwice, Poland

*Corresponding author

E-mail: joanna.polanska@polsl.pl (JP)

In this supplementary materials we present in more detail some issues related to aspects of GMM decompositions of protein mass spectra. These issues are presented in the three sections below.

In the first section we present detailed descriptions of successive steps of our algorithm for GMM decomposition of protein mass spectra.

In the second section we present some details of methods for generating and analyzing artificial datasets. We describe details of the elaborated scenarios for generation of artificial MS datasets and of comparisons of peak detection algorithms. We also describe tunable parameters of algorithms for peak detection and we show ROC curves plotted on the basis of changes of tunable parameters over the assumed limits.

In the third section we present comparisons between our algorithm for GMM decomposition of MS based on signal partition and the un-partitioned version of the GMM decomposition algorithm.

# Steps of the algorithm for GMM decomposition of spectra

In this subsection we present detailed descriptions of successive steps of our algorithm for GMM decomposition for MS signals.

## Baseline correction

We start the processing of the MS signal with the baseline correction (removal). In the proteomic MS data, the baseline can be quite precisely removed by using existing algorithms. In the implementation of our algorithms we are using the function “msbackadj” from Matlab Bioinformatics Toolbox [31].

Baseline correction is an important step of the algorithm. After removing the baseline there is no need to introduce wide Gaussian components for its modeling. Therefore, the algorithm can be oriented towards searching for narrow components corresponding to protein/peptide species in the analyzed samples. By cutting-off negative values we ensure that the baseline corrected signal is non-negative (important for its mixture modeling). A baseline corrected MS signal is the data for the subsequent steps of the algorithm.

## Peak detection

We start the processing of the baseline-corrected spectrum by launching a peak detection procedure. There are many algorithms for peak detection of the MS signals in the literature. In our implementation we use “mspeaks” function from Matlab Bioinformatics toolbox [31] (with the default settings), which provides estimates of both positions of spectral peaks and their widths (by the values of FWHH – full width at half high, returned by the “mspeaks” function). We divide each FWHH by the corresponding m/z value and we average over all detected peaks. The obtained value, proportional to the average coefficient of variation of Gaussian components, is used in the subsequent steps of the algorithm, for obtaining reliable estimates of sizes of splitter segments and segments, used in subsequent steps of the algorithm.

## Picking clear peaks and splitter-segments of the spectrum

We go through all of the detected peaks and we compute quality of each peak, given by the ratio of the peak height and maximum of heights of the neighboring lowest points of the MS signal. The neighboring right/left lowest points of the peak are minimum values of the spectral signal in between the peak’s m/z and the m/z of the next/previous peak. Then we apply a heuristic procedure for picking clear peaks (splitting peaks). The requirements are that (i) each of the splitting peaks is of sufficient quality, (ii) distances between successive splitting peaks satisfy demands given by specified parameters (neither too close nor to far one from another). We use average FWHH computed in the previous step to measure distances. For each clear peak (splitting peak) we define splitting-segment by cutting out a fragment of the MS signal around the splitting peak with suitably defined margins. For defining margins (sizes) of splitter-segments we again use average FWHH.

The heuristic method for picking a list of clear peaks is defined by the algorithm, whose pseudo-code is presented below:

**Input parameters:**

- *PL* - list of spectral peaks, elements of the list are: *p_no, p_mz, p_h, p_q* (peak number, peak m/z, peak height, peak quality)
- *pcv* - estimate of the coefficient of variation of peaks in the spectrum,
- *js, jl* – numbers, used for computing the distance to be either “skipped” or “looked up”.

**Auxiliary function: *find_dist*** (comment: distances between peaks computed with the help of *pcv*)

*p_end_no=****find_dist***(*p_start_no,PL,pcv,j*)

*p_run_no=peak_start_no;*

while 1

*p_run_no = p_run_no+1;*

if *p_mz(p_run_no)-p_mz(p_start_no)>j*pcv* p_mz(p_start_no)*

*p_end_no= p_run_no;*

break

endif

endwhile

Function: ***pick_clear_peaks***

list_of_clear_peaks = ***pick_clear_peaks*(***PL,pcv,js,jl***)**

*c_p_no=0* (comment: clear peak number)

*s_p_no=1* (comment: start peak number)

*e_p_no=1* (comment: end peak number)

while *not end of PL*

*s_p_no =* ***find_dist***(*s_p_no,PL,pcv,js*) (comment: skip)

*e_p_no =* ***find_dist***(*s_p_no,PL,pcv,jl*) (comment: look up)

*c_p_no = c_p_no+1*

*clear_peak(c_p_no) =* peak in the range *s_p_no ÷ e_p_no* with the highest quality

endwhile

Due to “skipping” fragments of spectra, the obtained clear peaks are not too close to each other. As seen in the “if” statement in the “find_dist” function the distance is measured using estimated average widths of peaks. In figure Fig. A we show a fragment of the average spectrum from [25] with 4 clear peaks, computed with the use of the above algorithm (*js=jl=4*).

**
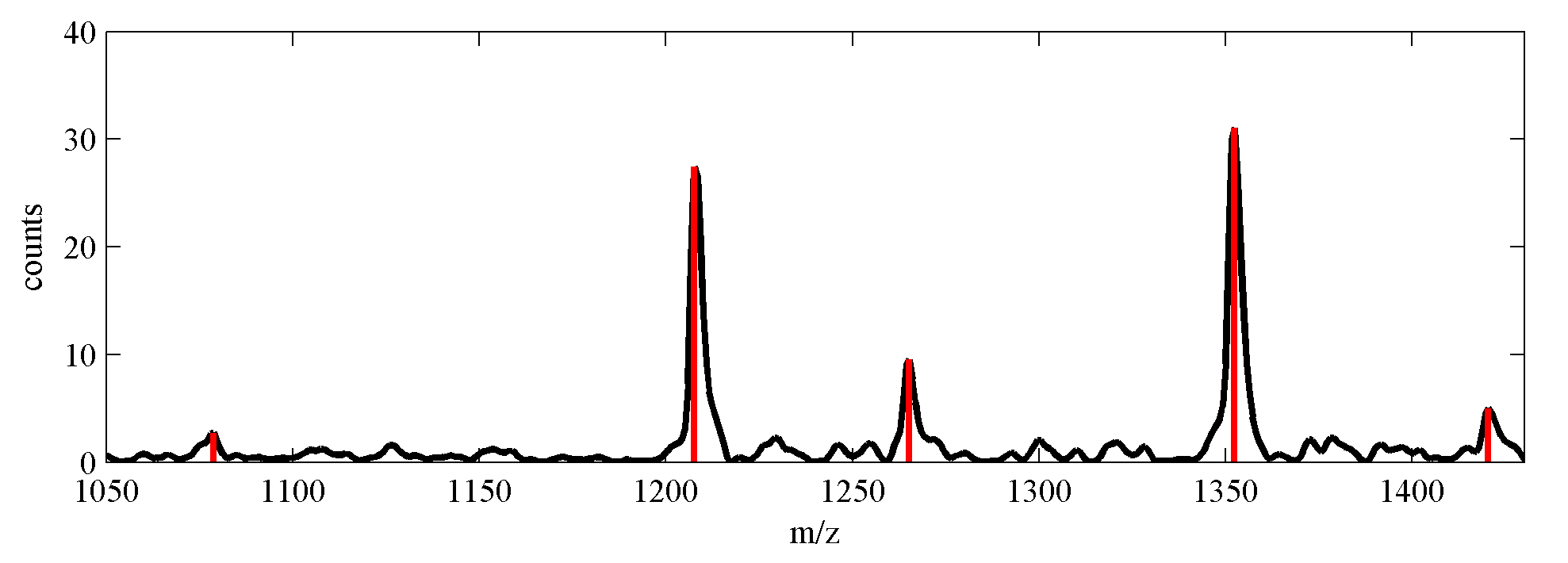
**

**Fig. A** Fragment of the average MS from the paper [25]. Picking clear peaks was done with the use of the algorithm described above with the (default) values *js=jl=4*. Clear peaks picked by the algorithm are marked by vertical red lines.

For each of the obtained clear peaks we define a splitter – segment by adding some margins on its left and right sides. Sizes of these margins are parameters of our algorithm, similarly to *js* and *jl*. Ranges of splitter-segments are computed by a function similar to “find_dist” defined above.

**
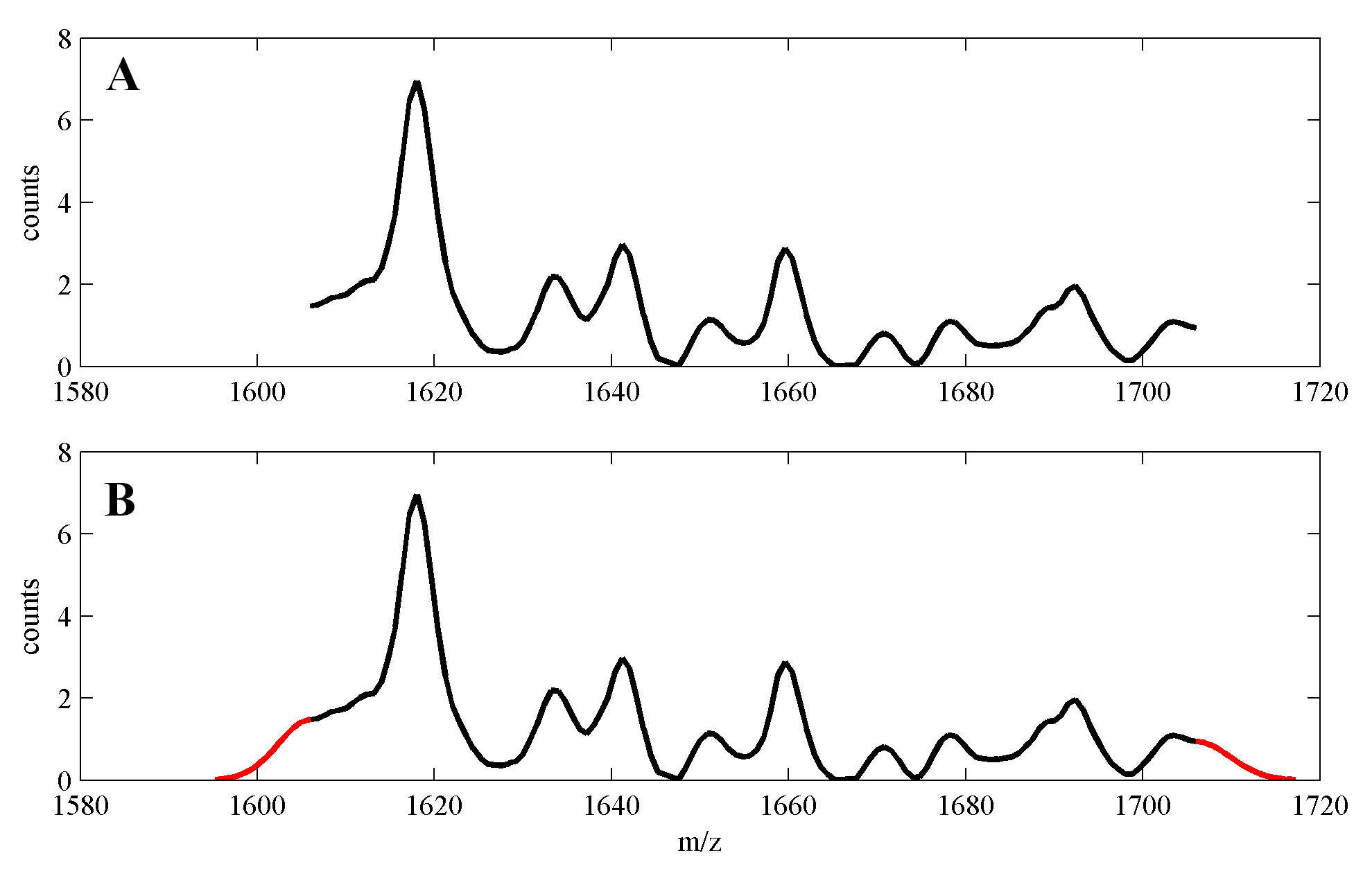
**

**Fig. B** Illustration of the “warping down” procedure. One of the splitter-segments computed for the average MS from the paper [25] is shown in black in the upper plot. In the lower plot this splitter-segment is “warped down” by adding two half Gaussian curves at both ends (shown in red).

MS signals at the borders of the splitter-segments can sometimes assume quite high values, which can lead to errors in mixture modeling related to boundary effects. In order to reduce this effect we have designed a procedure of “warping down” of the splitter-segments. “Warping down” is a heuristic procedure of augmenting the analyzed splitter-segments by adding artificial parts of the signal outside their borders. The added artificial parts are half Gaussian curves with widths defined by the *pcv* parameter multiplied by the m/z value. They are placed and scaled such that they assume values equal to signal values at the borders and vanish when the distance to the fragment increases. The “warping down” procedure is additionally illustrated in Fig. B, where in the upper plot (A) an example of a splitter-segment with ends assuming quite high values is shown. This example comes from the average spectrum from the paper [25]. In the lower plot (B) this splitter-segment (shown in black) is “warped down” by adding artificial parts, shown in red.

## GMM decompositions of splitter-segments, computing splitters

Splitter-segments signals are decomposed into Gaussian mixture models by using EM iterations. Initialization and execution of EM iterations is described in the subsequent paragraph “Execution of EM iterations”.

In the GMM model of the splitter-segment (Fig. 1 B from manuscript) components close to borders may be unreliable, due to boundary effects. However, components close to the splitting peak are assumed free from disturbances coming from boundaries. Therefore, we pick up components, such that distances between their means and the position of the splitting peak are less than three standard deviations. These components result in the splitter signal model (filled in red in Fig. 1 B from manuscript).

### Partitioning the spectrum into segments

We partition the baseline corrected MS spectrum into smaller fragments – segments, to later model each of them separately. Separated segment are obtained by subtracting splitter signals from the MS signal, as shown in Fig. 2 A, B from manuscript.

## GMM decompositions of segments

Each of the segments (splitter-segments) is decomposed into a Gaussian mixture model by using EM iterations. The procedure for initialization and execution of EM iterations is described in the forthcoming paragraph “Execution of EM iterations”.

### Execution of EM iterations

Parameters of the signal *y_n_*, $n_{\min}\leq n\leq n_{\max}$, (6)-(7) corresponding either to a splitter-segment or to a segment are $w_{k}=s\alpha_{k}$, $\mu_{k}$ and $\sigma_{k}$, *k=1,2,…K.* EM recursions assume the following form (similar to standard EM iterations for mixtures) [18, 22, 28]

$p(k|n)=\frac{\alpha_{k}f_{k}\left(x_{n},\mu_{k},\sigma_{k}\right)}{\sum_{\kappa=1}^{K} \alpha_{k}f_{\kappa}\left(x_{n},\mu_{\kappa},\sigma_{\kappa}\right)}$ (10)

$\alpha_{k}=\frac{\sum_{n=n_{min}}^{n_{max}} p(k|n)y_{n}}{\sum_{n=n_{min}}^{n_{max}} y_{n}}$ (11)

$\mu_{k}=\frac{\sum_{n=n_{min}}^{n_{max}} p(k|n)y_{n}x_{n}}{\sum_{n=n_{min}}^{n_{max}} p(k|n)y_{n}}$ (12)

$\sigma_{k}^{2}=\frac{\sum_{n=n_{min}}^{n_{max}} p(k|n)y_{n}(x_{n}-\mu_{k})^{2}}{\sum_{n=n_{min}}^{n_{max}} p(k|n)y_{n}}$ (13)

In (10) *p(k|n)* is the (estimated) conditional probability of measurements within the bin centered at *x_n_* belonging to (being generated by) *k*-th Gaussian component.

EM iterations (10)-(13) require specifying a method for setting initial values for parameters, $\alpha_{k}$, $\mu_{k}$ and $\sigma_{k}$. Appropriate initialization of EM is of critical importance for the convergence and quality of estimation. We are using here the algorithm for initialization of EM iterations, which applies dynamic programming partitions of the m/z values to estimate initial mixture parameters. The idea of partitioning an interval into bins, by using dynamic programming, with the aim of obtaining solution to some fitting or estimation problem was first formulated by Bellman [40] for approximating one-dimensional curves and then studied/used by other authors in many contexts, e.g. [41], [42]. Here the dynamic programming algorithm is planned such that the obtained bins should approximate ranges of m/z values corresponding to different components of the Gaussian mixture decomposition. Data from the separate bins are used to estimate initial values of weights, means and variances of Gaussian components for the EM algorithm. This algorithm shows advantages compared to other approaches. In the Matlab implementation of our GMM decomposition algorithm (available as S2 File), the function “dyn_pr_split.m” implements dynamic programming partition of the data range.

Some additional assumptions (modifications) must also be used for preventing a possible divergence of iterations. Mixture models fitted here to the MS signals are heteroscedastic (have unequal variances). For the case of unequal variances of components of the Gaussian mixture, the log-likelihood is unbounded [28, 43] which results in a possibility of encountering the divergence of EM iterations in computations. This problem is well known and there are several approaches published in the literature involving either a modification of the likelihood function [44] or introducing constraints on parameter values [45]. Here we prevent the divergence of EM iterations by simple constraint conditions concerning mixing proportions and component standard deviations.

We do not allow component standard deviations to fall below a certain threshold value. In other words we introduce the following additional operation in the iterations

$\sigma_{k}=\max\left(\sigma_{k},\sigma_{min}\right)$ (14)

The condition on mixing proportion involves removing too small components. If

$\alpha_{k}<\alpha_{min}$ (15)

then the component $\alpha_{k}$, $\mu_{k}$, $\sigma_{k}$ is removed from the current mixture model, and further EM iterations resume with re-indexed components, re-scaled mixing proportions and decremented *K=K-1*.

Threshold values $\alpha_{\min}$ and $\sigma_{\min}$ are parameters of the algorithm. The default value $\alpha_{\min}$ is assumed constant $\alpha_{\min}=10^{-3}$, while the value $\sigma_{\min}$ depends on m/z; it is computed as 0.5*(m/z)*(average coefficient of variation of Gaussian components).

The last issue to resolve is estimating the number of components in the mixture model of the splitter-segment or segment. In order to estimate the number of components, *K*, EM iterations described above are launched multiple times with different *K*. Then the value *K* is chosen on the basis of some criterion function. A widely used method for estimating *K* is application of the Bayesian information criterion (BIC) [28, 46], which combines values of the log likelihood and a (scaled) penalty for the number of components. Here, for the choice of *K* we use a penalty index, $I_{P}$ with the structure analogous to BIC, namely

$I_{P}=\text{Δ}+K\varepsilon$ (16)

In the above $\Delta$ is a scaled sum of absolute differences between the signal *y_n_* and its mixture model (the scaling factor is a value of TIC within the segment). We assume that $\varepsilon$ is a parameter to be chosen by the user. The default value is $\varepsilon=0.002$ (obtained on the basis of computational experiments).

The value of *K* is chosen on the basis of minimizing *I_P_* in (16) over a suitably defined range of changes of *K*. The choice of the range of changes of *K* in our algorithm follows from previously defined parameters of splitting MS by clear peaks. On the basis of default values of *js=jl=4* we expect that a splitter-segment contains approximately *10* components and a segment contains *4-8* components. Due to possible errors in these estimates we add some margins, which results in the range *5≤K≤18* for splitter-segments and *2≤K≤15* for segments.

## Post-processing of the GMM model parameters

When GMM modeling is used for peak detection in MS signals, additional step of post-processing of the mixture model is necessary for improving its efficiency. Post processing includes two procedures, rejection of components corresponding to noisy elements of spectral signals and merging components.

Spectral signals decomposed by using GMM, apart from shapes corresponding to protein/peptide species (peaks) can contain noise and residuals of baseline signals. Existence of these disturbing parts of spectral signals result in obtaining components in the GMM model, which do not correspond to true peaks. We are filtering out using a cut-off value for weights. This cut-off value is obtained by using a maximum a posteriori rule to the two-component mixture model fitted to the set of all weights of components in the Gaussian mixture model.

Due to the complexity of the mixture decomposition problem, it may happen that the obtained models contain components with similar values of means and variances (see examples of GMM decompositions in the Results section). For efficiency of the peak detection it might be reasonable to merge such similar components into one. The problem/need for merging similar Gaussian components was already encountered in practical applications of algorithms for decomposition of signals (datasets) into Gaussian mixtures. There are several papers devoted (or partly devoted) to methods of solving this problem e.g., [47].

Assume that there are two Gaussian components $\alpha_{1}\mu_{1}\sigma_{1}$ and $\alpha_{2}\mu_{2}\sigma_{2}$, which should be verified for being close enough to be replaced by one Gaussian component $\alpha,\mu,\sigma$. We compute differences $|\sigma_{1}-\sigma_{2}|$ and $|\mu_{1}-\mu_{2}|$. The merging threshold for standard deviations is assumed constant, equal to 0.05^-1^, and the merging threshold for means is denoted by

$\text{MZ}_{\text{thr}}=|\mu_{1}-\mu_{2}|$ (17)

We compute parameters$\alpha,\mu,\sigma$assuming that they follow from maximum likelihood estimates based on observations generated by the mixture model $\alpha_{1},\mu_{1},\sigma_{1},\alpha_{2},\mu_{2},\sigma_{2}$ which leads to estimates

$\alpha=\alpha_{1}+\alpha_{2}$ (18)

$\mu=\frac{\alpha_{1}}{\alpha}\mu_{1}+\frac{\alpha_{2}}{\alpha}\mu_{2}$ (19)

$\sigma^{2}=\frac{\alpha_{1}}{\alpha}\left(\mu_{1}^{2}+\sigma_{1}^{2}\right)+\frac{\alpha_{2}}{\alpha}\left(\mu_{2}^{2}+\sigma_{2}^{2}\right)-\mu^{2}$ (20)

# Scenarios for simulating and analyzing artificial datasets

Our scenarios for simulating and analyzing artificial datasets were similar to those previously applied by [32-34]. By using the VMS algorithm we have generated five datasets with different true numbers of protein (peptide) species, 100, 150, 200, 250 and 300. Each dataset contained 100 spectral lines (samples) defined over the same equally spaced grid of 10000 m/z points over the range 2000-10000 Da, with a step 0.8 Da (which apparently corresponded to low resolution spectrum). In each dataset we have first randomly created a list of artificial components (proteins). For each component we randomly draw (i) its mass by using a uniform distribution supported over the range 2000-10000 Da, (ii) a value of the prevalence of this component from a beta distribution with parameters a=1, b=0.2, (iii) the component abundance from the right – shifted by 100 counts log-normal distribution with mean equal to 5 and variance equal to 1. For each sample we then determine whether the component is present in the sample by a random Bernoulli trial with the probability defined by the assumed value of the protein prevalence. If the Bernoulli trial returns 1 we generate the peak’s intensity by drawing a random number distributed log-normally with mean *pe* equal to the component’s abundance and variance equal to $1.45\sqrt{pe}$. We then generate the position of the peak corresponding to the component (protein) using the normal distribution with mean $\mu$ equal to protein mass and standard deviation $\sigma=0.001\mu$, which reflects the misalignment of the peak’s position along the m/z axis between samples. The generated values of positions of peaks and their corresponding intensities are passed to the VMS algorithm, which generates an artificial spectrum containing the parts mentioned afore (the true spectral signal, baseline and noise). The default experiment parameters are used (mean initial velocity – 350 m/s, its standard deviation – 75 m/s, time between detector reads – 4e-9 s). Each synthetic spectrum includes a baseline and noise components. The baseline signal $y_{B}$ is modeled by using a formula with two exponential functions [32, 35]

$y_{B}=b\left(1\right)e^{-\frac{x}{b\left(2\right)}}-b(3)e^{-\frac{x}{b\left(4\right)}}$ (21)

where parameters *b(1)-b(4)* are chosen on the basis of empirical data. The random noise component (signal) is modeled by using discrete ARMA (auto-regressive, moving average) model with 1 AR term and 6 MA terms [32].

Analogously to the studies [32-34] in each of the datasets (100, 150, 200, 250 and 300 true peaks) we base detection of peaks on the mean spectral signals. Mean spectra are computed by averaging the spectral signals over the same values of m/z coordinates [12]. Hypothetical spectral peaks generated by MS-GMM, CWT and CROM are compared to the true spectral peaks. If the hypothetical spectral peak lays within the ±0.3% range of the true position of the spectral peak the true peak is considered detected. Otherwise the true spectral peak is considered missed. The margin for the error increases with increase in m/z, which is in concordance with the structure of MS, where peak widths are increasing with increasing m/z. The value ±0.3% used here is the same as in the algorithm applied in [34].

All algorithms have free, tunable parameters, which should be chosen prior to their application. We use F1 index (8) as base for tuning (optimizing) parameters of algorithms and we optimize performances of MS-GMM, CWT and CROM with respect to the average value of the F1 index over the ranges of their parameters analogously to [32-34].

In our algorithm MS-GMM we have adjusted one parameter, *MZ_thr_* (described earlier in the Methods section). Algorithms CWT and CROM include parameters, which can be tuned to data. For the CWT algorithm the adjustable parameters are Signal to Noise Ratio threshold (SNR), the scale range of the peak (peakScaleRange) and the minimal value of amplitude for the peak to be detected (ampTh). For the CROM algorithm the adjustable parameters are Signal to Noise Ratio threshold (SNR), a threshold for wavelet coefficients (W-threshold) and number of wavelets used in the transformation (L). Following recommendations of the authors and results from [32-34], for both algorithms we were tuning two parameters, namely SNR and peakScaleRange for CWT, and SNR and W-threshold for CROM. Values of the third parameter in both algorithms were set constant ampTh=0, L=10.

Optimal values of parameters of the algorithms were obtained by exhaustive searches through their parameter spaces, over the following ranges: 0.05 ≤ *MZ_thr_* ≤ 1 (MS-GMM); 1 ≤ SNR ≤ 5, 1 ≤ peakScaleRange ≤ 10 (CWT) and 10 ≤ SNR ≤ 115, 20 ≤ W-threshold ≤ 140 (CROM). Optimization was performed separately for each dataset, with 100, 150, 200, 250, 300 true peaks, and involved averaged values of the F1 index. The obtained optimal values of parameters are reported in table A.

**Table A.** Optimized values of parameters for algorithms MS-GMM, CWT and CROM

| **Algorithm** | **Parameters** | **Number of true peaks** | | | | |
| --- | --- | --- | --- | --- | --- | --- |
|  |  | **100** | **150** | **200** | **250** | **300** |
| **MS-GMM** | MZthr | 0.40 | 0.30 | 0.30 | 0.20 | 0.15 |
| **CWT** | SNR | 2 | 2 | 1 | 3 | 2 |
|  | PeakScaleRange | 7 | 5 | 5 | 2 | 2 |
| **CROM** | SNR | 55 | 40 | 100 | 115 | 115 |
|  | W-Threshold | 125 | 125 | 50 | 20 | 20 |

By using the algorithms with different values of their tunable parameters we were also able to estimate ROC curves, i.e., plots of sensitivity versus FDR, which are shown in Fig. C. In the plots in Fig. C, for all values of the true numbers of peaks, one can see the increase of the sensitivity, at the same values of FDR, obtained by our MS-GMM algorithm, when compared to CWT and CROM. Ranges of changes of FDR in all plots in Fig. C are significantly narrower than the full possible limit 0-1. This effect follows from the existence of mechanisms of estimation of numbers of peaks (components) and their filtration in all three compared algorithms.

**
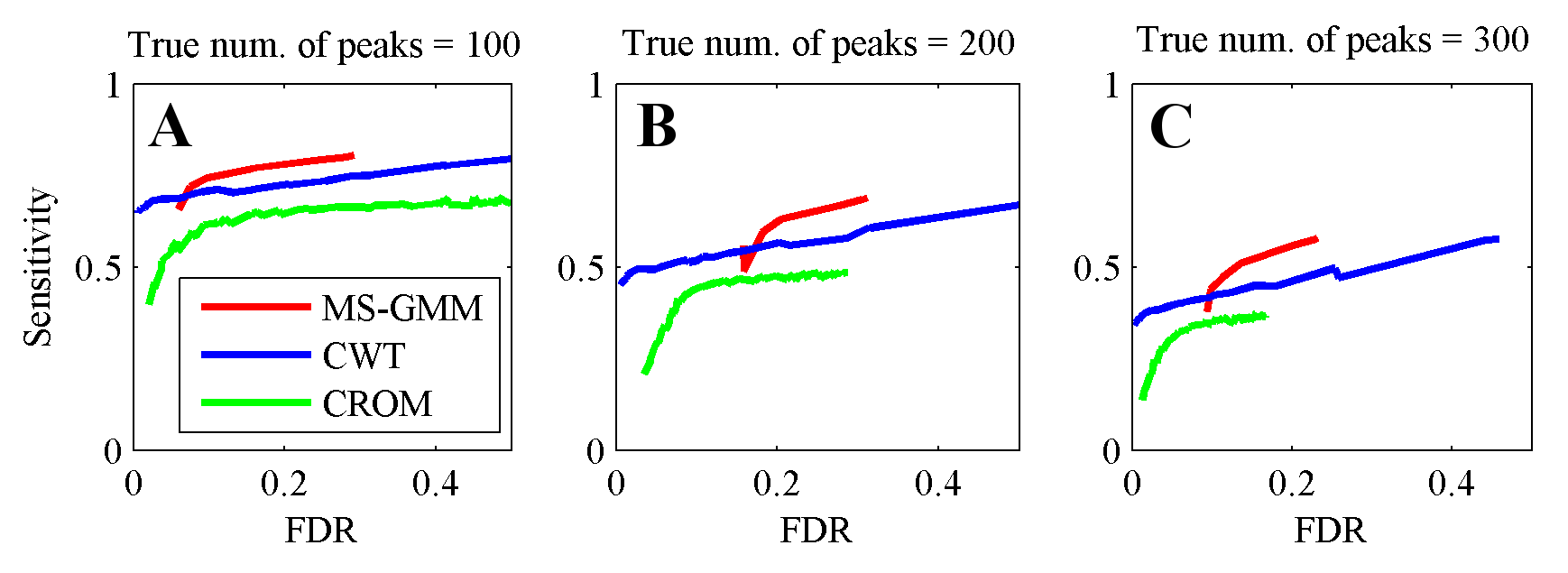
**

**Fig. C**ROC curves (plots of FDR versus sensitivity) for the compared algorithms MS-GMM, CWT and CROM, for three cases described by true numbers of peaks. **(A)** 100 true peaks, **(B)** 200 true peaks, **(C)** 300 true peaks. Colors: MS-GMM – red, CWT – blue, CROM – green.

# Comparisons between partitioned and un-partitioned versions of the GMM decomposition algorithms

In this subsection we illustrate in more detail some aspects of efficiency achieved by using the idea of partitioning the proteomic spectrum. We compare partitioned and un-partitioned versions of GMM decomposition algorithms.

According to our best knowledge, at present there is no software either publicly or commercially available, capable to perform GMM decompositions of MS at the whole spectra scale. In order to study the influence of partitioning on the computational aspects we have used our own software system written in Matlab environment in two modes with partitioning and without partitioning. The computations were performed for the average spectrum from the paper [25] and for one, randomly chosen, high resolution spectrum from the Aurum dataset. In both cases we used the same rules for the stopping criterion for EM iterations. Computational server used in obtaining GMM decompositions was the same as that described at the beginning of the Results section.

The computational experiment was designed as follows. In the first step the MS-GMM algorithm (the partitioned version) was applied with the default settings. Estimates of the GMM decomposition including the estimate *K* of the number of Gaussian components were computed. In the second step the un-partitioned GMM algorithms was applied. It was launched with the number of Gaussian components *K* obtained in the previous step. Initial values for m/z of components were obtained on the basis of positions of peaks detected by the CWT algorithm. Initial values of standard deviations of components were set on the basis of the average FWHH (computed by using CWT) and initial weights were set all equal.

## Computational times

The computational time for the average spectrum from [25] (approximately 10000 m/z values) was 95.3 seconds for the partitioned version of the algorithm and 1537.6 seconds for the un-partitioned version.

For the Aurum dataset spectrum T10467-Well A10_16783 (approximately 100000 m/z points) computational times were, 1379.2 seconds for the partitioned version of the algorithm and 280434.2 seconds for the un-partitioned version.

## Qualities of GMM decompositions

We first compare qualities of GMMs between version of the algorithm in terms of log likelihoods. For the average spectrum from [25] the partitioned version of the algorithm led to GM decomposition with the log likelihood L=‑4.662E+04, while the un-partitioned version led to GM with L=‑4.687E+04. For the Aurum dataset spectrum T10467-Well A10_16783 the partitioned version of the algorithm led to GM decomposition with the log likelihood L=‑1.159E+08, while the un-partitioned version led to GM with L=‑1.162E+08.

Additionally, for the Aurum spectrum T10467-Well A10_16783 we were also able to compare m/z positions (expectations) of the computed Gaussian components to true m/z values corresponding to peptides in the sample. We have computed relative errors for all “ground truth” peaks, for both versions of the algorithm using expression (9). For the partitioned version of the GMM algorithm median value of RE was equal to 0.000196 and for un‑partitioned version median was 0.000411. Interquartile ranges were, for MS_GMM equal to 0.000667 and for un‑partitioned version IQR=0.000433.

# Supplementary references

40. Bellman R. On the approximation of curves by line segments using dynamic programming. Commun ACM. 1961; 4: 284–295.

41. Jensen RE. A Dynamic Programming Algorithm for Cluster Analysis. Oper Res. 1969; 17: 1034–1057.

42. Jackson B. An algorithm for optimal partitioning of data on an interval. IEEE Signal Process Lett. 2005; 12: 105–108.

43. Kiefer J, Wolfowitz J. Consistency of the Maximum Likelihood Estimator in the Presence of Infinitely Many Incidental Parameters, Ann Math Statist. 1956; 27: 887-906.

44. Yao W. A profile likelihood method for normal mixture with unequal variance. J Stat Plan Inference. 2010; 140: 2089-2098.

45. Hathaway RJ. A constrained EM algorithm for univariate mixtures. J Stat Comput Simul. 1986; 23: 211-230.

46. Schwarz G. Estimating the Dimension of a Model. Ann Statist. 1978; 6: 461-464.

47. Runnalls AR. Kullback-Leibler Approach to Gaussian Mixture Reduction. IEEE Trans Aerosp Electron Syst. 2007; 43: 989–999.
